# Co-prescription of aripiprazole on prolactin levels in long-term hospitalized chronic schizophrenic patients with co-morbid type 2 diabetes: A retrospective clinical study

**DOI:** 10.3389/fpsyt.2023.1124691

**Published:** 2023-02-02

**Authors:** Xuebing Liu, Xianzhi Sun, Lu Li, Kuan Zeng, Yi Li, Yujun Gao, Jun Ma

**Affiliations:** ^1^Department of Psychiatry, Wuhan Mental Health Center, Wuhan, China; ^2^Wuhan Hospital for Psychotherapy, Wuhan, China; ^3^Department of Psychiatry, Renmin Hospital of Wuhan University, Wuhan, China

**Keywords:** schizophrenia, aripiprazole, prolactin, type 2 diabetes, long-term hospitalized, chronic

## Abstract

**Background:**

One of the most frequent side effects of atypical antipsychotics is hyperprolactinemia (HPRL), and metformin or aripiprazole co-prescription is regarded as an effective therapy option for reducing prolactin (PRL) levels. However, whether either of the two drugs can reduce PRL levels in patients with long-term hospitalized chronic schizophrenia with co-morbid type 2 diabetes (T2DM) has not been adequately reported.

**Methods:**

In our study, long-term hospitalized chronic schizophrenia patients with co-T2DM who were prescribed olanzapine or risperidone as the primary antipsychotic medication were enrolled. A total of 197 of these cases with co-prescribed aripiprazole were set up as the study group (co-Ari group), and the other 204 cases without co-prescribed aripiprazole were set up as the control group (non-Ari group). The two groups’ variations in each target parameter were compared, and the variables affecting PRL levels were examined.

**Results:**

Compared to the non-Ari group, fasting blood glucose (FBG), blood uric acid (UA), total cholesterol (TC), triglyceride (TG), and low-density lipoprotein cholesterol (LDL-C) levels were significantly higher in the co-Ari group, but there was no difference in PRL levels. Co-prescribing aripiprazole had no impact on PRL levels in all patients with co-T2DM, and aripiprazole dose had no impact on PRL levels in the clinical subgroup of the co-Ari group.

**Conclusion:**

Aripiprazole not only worsened the severity of index disturbances associated to metabolism in long-term hospitalized chronic schizophrenia patients with co-T2DM on metformin-based hypoglycemic medications but also failed to lower PRL levels.

## 1. Introduction

The most significant and fundamental form of treatment for schizophrenia is antipsychotic medication, as we all know. And one of the most prevalent and common adverse drug reactions to antipsychotic medications is hyperprolactinemia (HPRL) (1), but it is believed to have decreased with the broad and extensive clinical prescribing of second-generation antipsychotics as opposed to traditional antipsychotics (2). However, the real clinical situation may not be as optimistic as perceived. According to a significant Chinese study, the prevalence of HPRL in hospitalized schizophrenia patients is up to 61.3% and is similar in men and women (3). Among the many atypical antipsychotics, risperidone is considered to have one of the most significant effects on elevating prolactin (PRL) levels in psychiatric patients, and the PRL- increasing pharmacological effects of olanzapine should not be underestimated (1). A study from the Chinese province of Taiwan found that olanzapine can cause high levels of PRL (up to 51.6%) (4). What’s more, risperidone and olanzapine are two of the top-ranked antipsychotic drugs in terms of prescription rates, according to reports of prescribing patterns for psychiatric patients from around the world, including China (5–8). It is therefore not surprising to observe that HPRL brought on by atypical antipsychotics, such as risperidone and olanzapine, continue to be widespread and require proper clinical attention.

Long-term exposure to excessive PRL levels is frequently linked to an increased risk of sexual dysfunction (9), weight gain (10), cardiovascular disease (11), osteoporosis (12), and even cancer (13). In order to decrease the risk of adverse events and accidents in psychiatric patients and to promote patient compliance with treatment, it is vital for psychiatrists to find effective ways to diminish or neutralize antipsychotic-induced HPRL. In clinical practice, metformin is a frequently used hypoglycemic drug that has been shown to have pharmacological effects in attenuating antipsychotic-induced HPRL (14), but the specific mechanism of action is uncertain. It has been found that metformin can cross the blood-brain barrier and appears at higher levels in the pituitary gland than in other brain tissues (15). This suggests that metformin may have a local interaction with PRL-secreting cells in the pituitary gland to suppress elevated PRL levels. Distinguishing from metformin, as a novel antipsychotic with “PRL-sparing” effects (16, 17), aripiprazole has an partial agonistic effect on D_2_, as well as a partial agonistic effect on 5-HT_1A_ and/or an antagonistic effect on 5-HT_2A_ that exerts a PRL-lowering effect (18, 19). As a result, co-prescription of metformin or aripiprazole is considered an effective treatment option to reduce or alleviate HPRL caused by psychiatric drugs (20, 21). Unfortunately, the results of these studies or the specific recommendations given are for the general population with schizophrenia rather than for the group of long-term hospitalized chronic schizophrenia people with co-type 2 diabetes (T2DM).

We discovered by chance that aripiprazole did not lower PRL levels in a subclinical group of schizophrenic patients with co-T2DM in our earlier study when we examined the factors influencing PRL levels in chronically schizophrenic patients with long-term hospitalization for co-T2DM (22). However, due to the study’s limited sample size of aripiprazole-prescribed cases, statistical efficacy was compromised, leaving the conclusions without any useful advice for clinical action. The purpose of this study was to clarify the specific effects of aripiprazole on the PRL levels in co-T2DM schizophrenic patients, to fill in the gaps of the aforementioned study, to provide a reasonable and effective approach to the specific prescription, and to increase the sample size of this clinical subgroup.

## 2. Materials and methods

### 2.1. Subjects

A total of 197 schizophrenic patients with long-term hospitalization and co-T2DM who were hospitalized at Wuhan Mental Health Center and Suzhou Guangji Hospital from June 2015 to August 2022 with co-prescription of aripiprazole were included as the study group in this study.

#### 2.1.1. Inclusion criteria

(1) Meet the criteria for the diagnosis of schizophrenia in the QQInternational Classification of Diseases 10th Revision (ICD-10).

(2) Age range of 25–70 years old, male and female cannot be restricted.

(3) The duration of psychiatric illness was 6 years or more, and there were no adjustments in the dose or type of antipsychotic medication in the 2 months before collection of the target data.

(4) The duration of continuous uninterrupted inpatient treatment is not less than 2 years.

(5) The antipsychotic prescribed during hospitalization was olanzapine in combination with aripiprazole or risperidone in combination with aripiprazole, and the doses of the three antipsychotics involved were not limited.

(6) All enrolled patients were co-T2DM and were treated with oral hypoglycemic agents for glycemic control. Additionally, diabetes mellitus did not last less than 1 year.

#### 2.1.2. Exclusion criteria

Exclude bipolar disorder, major depressive disorder, personality disorders, psychiatric disorders due to epilepsy, intellectual developmental disorders, psychiatric disorders due to somatic disorders, and other psychiatric disorders other than schizophrenia. Those with type 1 diabetes (T1DM) and T2DM who need extra exogenous insulin for glycemic control should be excluded. Patients with severe physical comorbidities were also disqualified, including those with cerebrovascular disease, severe heart disease, somatic dysfunction, and other conditions that limit free movement and affect executive function, as well as those with polycystic ovary syndrome and pituitary tumors that affect PRL levels.

In the course of gathering study group cases, we included 204 schizophrenic patients with co-T2DM as the control group, which had the same inclusion and exclusion criteria as the study group, except that aripiprazole was not prescribed.

This study was reviewed and approved by the Ethics Committee of Wuhan Mental Health Center.

### 2.2. Research design

The study design was a two-center retrospective case-control study. We studied long-term hospitalized chronic schizophrenic patients with co-T2DM, with the group of patients co-prescribed with aripiprazole as the study group (co-Ari group) and the group of patients not co-prescribed with aripiprazole as the control group (non-Ari group), comparing the differences between the two clinical subgroups in terms of common demographic data and general clinical data, especially in terms of PRL levels. The factors influencing the PRL levels in the study group were also analyzed.

We extracted demographic information and general clinical data from the electronic case systems of the two centers for cases meeting the inclusion criteria for the study and control groups, including age, gender, educational background, body weight (BW), abdominal circumference (AC), body mass index (BMI), duration of psychiatric illness and age of onset, length of stay in the hospital, antipsychotic type and dose, glucose-lowering drug type and dose, fasting blood glucose (FBG), renal function [namely: blood urea nitrogen (BUN); blood creatinine (CRE); blood uric acid (UA)], blood lipids [namely: total cholesterol (TC); triglyceride (TG); low-density lipoprotein cholesterol (LDL-C); high-DL-C (HDL-C)], and PRL. The above indicators were recorded in a self-made spreadsheet.

We defined patients with continuous and uninterrupted hospitalization of greater than or equal to 2 years from the first day of the current hospitalization as long-term hospitalized patients. The time point at which the target parameters were tested and extracted for all samples was required to no history of antipsychotic drug type and dose adjustment and exogenous insulin supplementation in the 2 months before that time. All parameters involved that require the testing of venous blood were measured using morning fasting venous blood as the specimen. BW, AC, BMI were also measured and calculated values obtained in the morning fasting state.

### 2.3. Data analysis

The obtained continuous variables that fit the normal distribution were expressed as mean and standard deviation, and categorical variables were expressed as counts (percentages). First, we used independent samples *t*-tests or chi-square tests to compare the differences between the study and control groups for each target parameter. Secondly, we used Pearson’s correlation analysis to obtain parameters related to PRL levels for all included samples (a total of 401 participants). Thirdly, a multiple linear regression model was constructed to analyze the factors influencing PRL levels in the total sample size. Finally, a second multiple linear regression model was constructed for the study group sample to analyze the factors influencing PRL levels in this clinical subgroup. All *P*-values were two-tailed, and the significance level was <0.05. Statistical analyses were performed using SPSS 27 (SPSS, Inc., Chicago, IL, USA).

## 3. Results

### 3.1. Differences in target parameters between the two clinical subgroups

In patients prescribed olanzapine, the average daily drug dose of olanzapine was (12.86 ± 5.42) mg, and the average daily drug dose of risperidone was (4.10 ± 1.38) mg. The average daily dose of aripiprazole in the co-Ari group was (13.96 ± 3.75) mg. FBG, UA, TC, TG, and LDL-C levels were significantly higher in the study group compared to the control group (*t* = 2.00, *p* = 0.046; *t* = 2.17, *p* = 0.030; *t* = 2.50, *p* = 0.013; *t* = 3.81, *p* < 0.001; respectively), but there was no difference in PRL levels (*t* = 0.56, *p* = 0.579) (Table 1).

**TABLE 1:** Demographic and general clinical data.

Index	Total patients (*n* = 401)	Co-Ari (*n* = 197)	Non-Ari (*n* = 204)	t/χ^2^	*p*-value
Age–years	46.93 ± 10.33	47.18 ± 10.03	46.69 ± 10.63	0.48	0.634
Gender–*n* (%)				1.20	0.274
Female	259, 64.59%	122, 61.93%	137, 67.16%		
Male	142, 35.41%	75, 38.07%	67, 32.84%		
Schizophrenia duration–years	18.55 ± 10.03	18.38 ± 10.16	18.72 ± 9.93	−0.33	0.739
Onset age–years	28.38 ± 8.20	28.80 ± 8.23	27.97 ± 8.17	1.01	0.314
Length of hospital stays–years	4.59 ± 1.23	4.55 ± 1.13	4.63 ± 1.32	−0.65	0.519
Antipsychotic drugs–*n* (%)				0.45	0.505
Olanzapine	254, 63.34%	128, 64.97%	126, 61.76%		
Risperidone	147, 36.66%	69, 35.03%	78, 38.23%		
Diabetes duration–years	4.46 ± 1.97	4.32 ± 1.89	4.60 ± 2.04	−1.42	0.157
Anti-glycemic drugs–*n* (%)				3.07	0.080
Metformin alone	75, 32.86%	30, 15.23%	45, 22.06%		
Co-metformin	326, 81.30%	167, 84.77%	159, 77.94%		
Metformin dosage–g	1.14 ± 0.36	1.15 ± 0.37	1.13 ± 3.43	0.63	0.532
Educational background–*n* (%)				0.07	0.787
Junior school and below	245, 61.10%	122, 61.93%	129, 63.24%		
High school and above	156, 38.90%	75, 38.07%	75, 36.76%		
FBG–mmol/L	7.41 ± 2.74	7.69 ± 2.68	7.14 ± 2.77	2.00	0.046*
BUN–mmol/L	3.91 ± 3.17	4.21 ± 3.61	3.63 ± 2.66	1.83	0.068
CRE–mmol/L	64.18 ± 20.89	65.35 ± 22.09	63.05 ± 19.66	1.10	0.271
UA–mmol/L	412.54 ± 121.43	417.17 ± 120.67	408.07 ± 122.29	0.75	0.454
TC–mmol/L	4.54 ± 1.06	4.66 ± 0.96	4.43 ± 1.13	2.17	0.030*
TG–mmol/L	3.13 ± 2.35	3.43 ± 2.45	2.85 ± 2.22	2.50	0.013*
LDL-C–mmol/L	2.25 ± 0.84	2.41 ± 0.86	2.10 ± 0.79	3.81	<0.001*
HDL-C–mmol/L	0.99 ± 0.25	0.98 ± 0.26	1.01 ± 0.23	−0.27	0.091
BMI–kg/m^2^	22.89 ± 9.20	23.25 ± 10.56	22.28 ± 7.65	1.35	0.177
BW–kg	60.49 ± 11.05	60.66 ± 11.05	60.32 ± 11.07	0.31	0.755
AC–cm	80.12 ± 8.74	80.23 ± 9.57	79.19 ± 8.02	1.18	0.054
PRL–ng/mL	31.58 ± 26.59	32.33 ± 25.77	30.86 ± 27.40	0.56	0.579

Co-Ari, co-prescribed aripiprazole, i.e., study group; non-Ari: non-prescribed aripiprazole, i.e., control group; FBG, fasting blood glucose; BMI, body mass index; BUN, blood urea nitrogen; CRE, blood creatinine; UA, blood uric acid; TC, total cholesterol; TG, triglyceride; LDL-C, low-density lipoprotein cholesterol; HDL-C, high-density lipoprotein cholesterol; BW, body weight; AC, abdominal circumference; PRL, prolactin. *P < 0.05.

### 3.2. Factors associated with PRL levels for all included patients

For all included co-T2DM schizophrenia patients, age (*r* = 0.01, *p* = 0.017), schizophrenia duration (*r* = 0.17, *p* = 0.001), prescription olanzapine (*r* = 0.11, *p* = 0.035), prescription risperidone (*r* = −0.03, *p* = 0.035), and LDL-C levels (*r* = 0.27, *p* < 0.001) were positively associated with PRL levels, while, prescription metformin (*r* = −0.11, *p* = 0.048), prescription aripiprazole (*r* = −0.01, *p* = 0.009), FBG levels (*r* = −0.09, *p* = 0.041), and UA levels (*r* = −0.14, *p* = 0.007) were positively associated with PRL levels (Table 2).

**TABLE 2:** Factors associated with PRL levels in patients with schizophrenia co-type 2 diabetes (T2DM).

Characteristic	*n* = 401	
	*r*	*p*-value
Age–years	0.01	0.017*
Schizophrenia duration–years	0.17	0.001*
Onset age–years	−0.20	0.060
Length of hospital stays–years	−0.01	0.794
Diabetes duration–years	0.03	0.623
Educational background	0.07	0.184
Metformin	−0.11	0.048*
Aripiprazole	−0.03	0.009*
Olanzapine	0.11	0.035*
Risperidone	0.01	0.035*
FBG–mmol/L	−0.09	0.041*
BUN–mmol/L	−0.04	0.401
CRE–mmol/L	−0.06	0.257
UA–mmol/L	−0.14	0.007*
TC–mmol/L	0.12	0.018
TG–mmol/L	0.01	0.791
LDL-C–mmol/L	0.27	<0.001*
HDL-C–mmol/L	0.03	0.497
BMI–kg/m^2^	0.04	0.455
BW–kg	0.12	0.059
AC–cm	0.01	0.809

FBG, fasting blood glucose; BMI, body mass index; BUN, blood urea nitrogen; CRE, blood creatinine; UA, blood uric acid; TC, total cholesterol; TG, triglyceride; LDL-C, low-density lipoprotein cholesterol; HDL-C, high-density lipoprotein cholesterol; BW, body weight; AC, abdominal circumference; PRL, prolactin. *P < 0.05.

### 3.3. Factors influencing PRL levels in all included patients

We constructed a multivariate linear model with PRL level as the dependent variable and the above clinical parameters associated with PRL level as independent variables. The three variables involved in the model, aripiprazole, olanzapine, and risperidone, were defined as dichotomous variables (0 = prescribed, 1 = unprescribed). Age (*B* = −0.61, *t* = −3.64, *p* = 0.021), prescription metformin (*B* = −0.12, *t* = −1.04, *p* = 0.017), and FBG (*B* = −1.71, *t* = −3.42, *p* = 0.001) levels were protective factors for HPRL, whereas prescription olanzapine (*B* = 2.11, *t* = 1.05, *p* = 0.036) and risperidone (*B* = 7.21, *t* = 2.72, *p* = 0.007) were risk factors for HPRL (Table 3).

**TABLE 3:** Influencing factors of prolactin (PRL) levels in include patients with type 2 diabetes (T2DM): multiple linear regression model.

	Coefficients	Std. error	*t*	*p*-value	95% CI for EXP (*B*)	
	*B*				Lower	Upper
Constant	65.63	9.76	6.72	<0.001	46.44	84.82
Age–years	-0.61	0.17	-3.64	0.021*	-0.94	-0.28
Schizophrenia duration–years	1.05	0.18	5.94	0.081	0.70	1.39
Metformin–g	-0.12	0.04	-1.04	0.017*	-0.21	0.00
Aripiprazole	-2.58	2.57	-1.00	0.317	-7.63	2.48
Olanzapine	2.11	1.46	1.05	0.036*	1.12	1.89
Risperidone	7.21	2.65	2.72	0.007*	1.99	12.42
FBG–mmol/L	-1.71	0.50	-3.42	0.001*	-2.70	-0.73
UA–mmol/L	-0.04	0.01	-3.38	0.051	-0.06	-0.02
LDL-C–mmol/L	2.20	1.55	1.41	0.158	-0.86	5.25

FBG, fasting blood glucose; UA, blood uric acid; LDL-C, low-density lipoprotein cholesterol. *P < 0.05.

### 3.4. Factors influencing PRL levels in clinical subgroups prescribed aripiprazole

In the clinical subgroup co-prescribed with aripiprazole, we constructed a multiple linear regression model again with PRL levels as the dependent variable and parameters associated with PRL levels as independent variables. Age (*B* = −0.95, *t* = −2.93, *p* = 0.014), prescription metformin (*B* = −0.11, *t* = −0.89, *p* = 0.015), FBG (*B* = −3.63, *t* = −3.71, *p* < 0.001) levels, and UA (*B* = −0.07, *t* = −3.18, *p* = 0.002) levels were protective factors for HPRL, whereas prescription risperidone (*B* = 2.32, *t* = 3.23, *p* = 0.032) were risk factors for HPRL (Table 4).

**TABLE 4:** Influencing factors of prolactin (PRL) levels in clinical subgroups prescribed aripiprazole: multiple linear regression model.

	Coefficients	Std. error	*t*	*p*-value	95% CI for EXP (*B*)	
	*B*				Lower	Upper
Constant	39.61	25.34	5.51	<0.001	89.44	189.78
Age–years	-0.95	0.33	-2.93	0.014*	-1.59	-0.31
Schizophrenia duration–years	0.79	0.34	2.37	0.120	0.13	1.46
Metformin–g	-0.11	0.21	-0.89	0.015*	-0.15	0.01
Aripiprazole–mg	-0.28	0.91	-0.31	0.759	-2.08	1.52
Olanzapine–mg	-0.76	0.43	-1.74	0.084	-1.62	0.10
Risperidone–mg	2.32	1.97	3.23	0.032*	0.73	8.21
FBG–mmol/L	-3.63	0.98	-3.71	<0.001*	-5.57	-1.69
UA–mmol/L	-0.07	0.02	-3.18	0.002*	-0.11	-0.03
LDL-C–mmol/L	0.51	2.78	0.18	0.856	-4.99	6.01

FBG, fasting blood glucose; UA, blood uric acid; LDL-C, low-density lipoprotein cholesterol. *P < 0.05.

## 4. Discussion

According to us, this may be the only study to date to clarify whether aripiprazole can reduce PRL levels in schizophrenia patients with co-T2DM. The main findings of this study were that co-prescribing aripiprazole not only had no clinical value in reducing PRL levels in the target population of our study but also increased the severity of metabolic disorders compared to the clinical subgroup without co-prescribing aripiprazole. Our secondary findings also include: (1) in the group of schizophrenic patients with co-T2DM, higher age, prescription metformin, and higher FBG levels were protective factors for HPRL, and prescription olanzapine and risperidone were risk factors for HPRL, but aripiprazole did not affect PRL levels. (2) In the subclinical group with co-prescribed aripiprazole, higher age, metformin dose, and higher FBG and UA levels were protective factors for HPRL, and risperidone dose was a risk factor for HPRL, but aripiprazole dose had also no effect on PRL levels.

There are many clinical studies on the effectiveness of aripiprazole in reducing antipsychotic-induced HPRL (23–25). In China, it is relatively uniform and widely accepted that co-prescribing aripiprazole at doses less than 5 mg is the most effective and optimal prescribing regimen (14, 26). However, one meta-analysis gave differing conclusions, such as Zhang et al. (27) who included 53 randomized controlled double-blind studies and found that either adjuvant less than 5 mg or greater than 10 mg of aripiprazole was the best regimen to control antipsychotic-induced HPRL. In contrast to the above reports, a study from India found that the percentage reduction in PRL levels did not correlate with the specific dose of aripiprazole (28). A multicenter, open-label, prospective study from Korea also reported that administration of the maximum dose of co-prescribed aripiprazole (30 mg/day) similarly achieved a reduction in antipsychotic-induced HPRL (29). These above studies may suggest that the PRL-sparing effect of aripiprazole may not be dependent on the dose of the drug. Although there is a wide range of opinions about which dose of aripiprazole is optimal for improving HPRL, the conclusion that aripiprazole can reduce antipsychotic-induced HPRL is relatively uniform and clear. Puzzlingly, our findings all differ from the above studies in that we found no actual clinical value of co-prescribing aripiprazole for lowering PRL levels in the schizophrenia group with co-morbid T2DM. We speculate that this may be related to the more specific study population we enrolled in or the narrower range of aripiprazole doses (10–20 mg/day) that the study population was prescribed.

As a basic and primary therapeutic agent for T2DM, metformin also unsurprisingly showed a large prescription rate for controlling patients’ blood glucose levels in the schizophrenia group included in our study, while its other significant pharmacological effect is its use for controlling antipsychotic-induced HPRL (14, 20, 27). There is similar controversy and uncertainty regarding the optimal dose of metformin for the treatment of antipsychotic-induced HPRL. The expert consensus from China and clinical studies from Poland both conclude that high doses of metformin (2.55–3.0 g/day) are effective in reducing antipsychotic-derived HPRL (14, 30), but this dose exceeds the maximum daily dose limit (maximum 2 g/day) given in the metformin instructions, which may introduce other metformin-derived adverse drug reactions and ethical issues, and therefore should not be used as a routine clinical treatment regimen. A meta-analysis reported that metformin doses below 1 g/day were also effective in reducing PRL levels in patients with HPRL induced by atypical antipsychotics (27). In our study, the conventional metformin dose (1–2 g/day) used to treat diabetes mellitus in schizophrenia with co-morbid T2DM also had the same function of lowering PRL levels and showed a negative dose-dependence of metformin dose and PRL levels. Whether the reason for this phenomenon is related to the co-prescription of aripiprazole, resulting in a dose shift of metformin to lower PRL levels, is a question that deserves further investigation. And whether aripiprazole is competing with metformin for the failure of targets that inhibit the synthesis and/or release of PRL and thus losing its function in reducing the utility of PRL levels is likewise a question that needs to be further answered.

In the present study, although the function of lowering PRL levels was lost, the co-prescription of aripiprazole exacerbated the severity of abnormal metabolic markers in the included patients, although aripiprazole is considered to be one of the antipsychotics with the least metabolic adverse effects (31–33). In contrast to the more common extrapyramidal adverse effects of first-generation antipsychotics, atypical antipsychotics exhibit more prominent abnormalities in metabolic indicators (34). A study from Hong Kong, China, reported that the combination of multiple antipsychotics increased the risk of abnormal metabolic parameters associated with cardiovascular disease in patients with schizophrenia spectrum disorders (35). Another study reported a higher incidence of metabolic syndrome and lipid markers of insulin resistance in patients receiving antipsychotic polypharmacy compared to those receiving antipsychotic monotherapy (36). In contrast to our study, the participants we included were co-prescribed aripiprazole, which is thought to have no or minimal effect on metabolic indices, and two studies even reported that adjunctive use of 5–20 mg/day of aripiprazole improved metabolic disturbances, while the original atypical antipsychotic dose was maintained (37–40). However, a review of systematic reviews concludes that this possible protective effect of aripiprazole needs to be further elaborated by more robust studies (41), because longer-term observations and studies have found that the severity of metabolic adverse effects of aripiprazole is not superior to that of antipsychotics such as risperidone and quetiapine (42, 43). This is consistent with the results of our study, which found that long-term hospitalized chronic schizophrenia patients co-prescribed with aripiprazole exhibited metabolic abnormalities of even worse severity.

In the secondary findings, we found that older age was a protective factor for HPRL, and one study also found that antipsychotic-derived high levels of PRL decline with age in patients with schizophrenia (44), which may be attributed to the fact that the gonads shrink with age. Higher FBG levels were also a protective factor for HPRL, which is inconsistent with previous findings (45), and in our opinion may be related to the more aggressive addition of glucose-lowering agents represented by metformin for those patients with poorly controlled blood glucose levels. Risperidone remained an important contributor to HPRL, which was the same as the previous findings (1). And higher UA levels were a protective factor for HPRL, which was consistent with our previous report (22).

## 5. Conclusion

In conclusion, aripiprazole not only worsened the severity of index disturbances associated to metabolism in long-term hospitalized chronic schizophrenia patients with co-T2DM on metformin-based hypoglycemic medications but also failed to lower PRL levels.

## Data availability statement

The raw data supporting the conclusions of this article will be made available by the authors, without undue reservation.

## Ethics statement

This study was reviewed and approved by the Ethics Committee of Wuhan Mental Health Center. Written informed consent to participate was not required in accordance with institutional requirements and legislation.

## Author contributions

JM and YG made substantial contributions to conception and design of the study. XL and XS drafted the manuscript. LL had polished and re-edited the language and logic of the manuscript. KZ and YL were responsible for setting up and complement and modify the contents of the manuscript. JM gave final approval of the version to be published. All authors contributed to the article and approved the submitted version.
